# Talus bipartitus: a systematic review and report of two cases with arthroscopic treatment

**DOI:** 10.1007/s00167-017-4613-8

**Published:** 2017-06-28

**Authors:** Ruben Zwiers, Peter A. J. de Leeuw, Gino M. M. J. Kerkhoffs, C. Niek van Dijk

**Affiliations:** 1 Department of Orthopaedic Surgery, Academic Medical Center, University of Amsterdam, PO Box 22660, 1100 DD Amsterdam, The Netherlands; 2 Department of Orthopaedic Surgery, Flevoziekenhuis, Almere, The Netherlands; 3 Academic Center for Evidence Based Sports Medicine (ACES), Amsterdam, The Netherlands; 4 Amsterdam Collaboration for Health and Safety in Sports (ACHSS), Amsterdam, The Netherlands

**Keywords:** Ankle, Talus bipartitus, Arthroscopy

## Abstract

**Purpose:**

The aim of this study was to provide a literature review on talus bipartitus and to introduce an arthroscopic treatment option.

**Methods:**

A systematic review of published case reports and small case series was performed. Medline, Embase, CINAHL, Google Scholar and Web of Science databases were searched for relevant publications. In addition, three cases of talus bipartitus treated in our institute were discussed.

**Results:**

Eleven articles were identified, reporting on 23 patients, of whom one patient had a bilateral talus bipartitus. Fourteen were males (61%). The median age at presentation was 15.5 years (IQR 14–24.3). In 21 of the symptomatic cases (96%), the patient experienced ankle pain, and 13 had a restricted range of motion (54%). In our institution, two patients were treated arthroscopically and had excellent short- and long-term outcomes.

**Conclusion:**

Talus bipartitus is a rare anatomical anomaly. Symptoms are characterized by pain and restricted subtalar motion in young patients. Surgical treatment is focused on either fixation or excision of the bony fragment. Our two cases have demonstrated that an arthroscopic approach can be a safe and effective treatment option in patients with a symptomatic talus bipartitus.

**Level of evidence:**

IV.

## Introduction

Talus bipartitus is a rare bony anomaly of the talus. It is defined as two non-fused talar fragments, with the posterior fragment located at the level of the posterior talar process. Strehle in his dissertation on talar anomalies described the first case in 1928 [17]. Talus bipartitus needs to be distinguished from the more common os trigonum (or Shepherd fracture), which only includes the posterolateral tubercle of the posterior talar process [10]. Also it should be distinguished from the Cedell fracture, for both its location and its aetiology. The Cedell fracture is an avulsion of the posteromedial tubercle of the posterior talar process and is thought to occur due to tensile forces from the posterior tibiotalar ligament [2].

The prevalence of the talus bipartitus is unknown, and only a few case reports and small case series have been published. Little is known about its aetiology, clinical manifestations, treatment and outcome. Therefore, the purpose of this study was to provide an overview of the current literature. A systematic review of published case reports and small case series was performed. In addition, all patients that were treated over the last 15 years in our institution were reviewed. In this study, both the diagnostic process and the treatment of these patients will be described, including the long-term follow-up.

## Materials and methods

Medline and Embase databases were searched for relevant publications, using the following terms (until 1 December 2015): (Talus OR Talar OR Astragalus) AND (bipart* OR partit* OR secondary OR fragment) OR frontal split. In addition, the CINAHL, Google Scholar and Web of Science databases were searched for scientific papers, book chapters and abstracts. Reference lists of relevant articles were reviewed. Titles were screened on relevance, and subsequently, full-text articles were assessed. The search was limited to articles describing humans.

Studies were only included if at least one case of a talus bipartitus was described, no language restriction was applied. Data on aetiology, clinical presentation, imaging and treatment were extracted. Due to the small amount of data, the presentation of data was descriptive. In addition to the literature review, three patients with a symptomatic talus bipartitus treated in our institution over the last 15 years were presented. Collection of data for this study was approved by the local research ethics committee (W12_237#12.17.0270).

## Results

The literature search yielded 956 articles, of which 22 articles were selected for full-text screening. Of these, 11 articles were considered eligible. One article was added after screening literature lists on relevant papers. The included articles consisted of seven case reports [1, 3, 6, 15, 16, 18, 22], one case description in a dissertation [17] and four small case series [5, 9, 12, 13]. These articles reported on 23 patients, of whom one patient had a bilateral talus bipartitus. Fourteen of them were males (61%). The median age at presentation was 15.5 years (IQR 14–24.3). In 21 of the symptomatic cases (96%), the patient experienced ankle pain, mainly on weight bearing. Thirteen had a restricted range of motion (54%). Two cases were coincidently detected following an ankle trauma. Most of the patients had no history of ankle trauma. In nine cases (38%), radiological imaging showed some signs of subtalar- and or talocrural joint degeneration. The size of the osseous fragment and extent of joint involvement varied widely. Surgical treatment had been performed in 19 cases (90%). In 13 cases (68%), excision of the fragment was performed by open surgery. Of these, due to ongoing symptoms, in four patients a secondary surgical procedure was performed, three patients underwent a subtalar fusion, and in one patient, a pantalar fusion was performed. In one case, the primary surgical intervention was a subtalar fusion, and in five cases, fixation of the bony fragment was performed. Due to the variety of presentations and limited descriptions on the specific treatment, a profound comparison of the outcomes was not possible. An overview of the published cases is presented in Table 1.

**Table 1:** Overview of published cases

Study	Year	Age	Sex	Side	Complaints	Phys. exam	Traumatic	Duration	Imaging		Size	Treatment	Outcome	Follow-up
Strehle [1]	1928	14	F	Left	Pain during walking	–	No	8	X-ray	TB, incongruent joints	–	–	–	–
Weinstein [9]	1975	13	M	Right	Increasing pain lateral malleolus, radiating anteriorly	Restricted ROM TCJ and STJ	No	0.4	X-ray	TB	2.5 × 2 × 2 cm, 100% STJ	Excision	–	–
Schreiber [10]	1985	15	F	Left	Dull ankle pain over medial malleolus after exercise	Restricted. Dorsal flexion TCR, restr. ROM STJ, pain	–	4	X-ray, arthrography	TB, flattened talar dome	Both joints involved	–	–	–
Blauth [5]	1987	18	F	Left	Pain during activity	Restricted ROM AJ	No	2	X-ray	TB, sclerotic, congruent	1/3 talar dome, both joints involved	–	–	–
Griffet [7]	2003	15	F	Left	Intermittent ankle pain during activity	Normal	No	1	X-ray, MRI	TB, incongruent fragments both joints, degenerative, aberrant aspect calcaneus	Posterior 1/3 of talar body	Subtalar arthrodesis	Decrease pain, recurrence after 2 y, conservatively treated	2
Eichenbaum [14]	2010	18	M	Right	Increasing hindfoot pain	FROM TCJ, painful restricted ROM STJ	No	4	X-ray, CT	TB, degenerative changes	4 × 2 × 1.5 cm, 50% post STJ	Excision + subtalar arthrodesis	Pain-free activity	1
		16	M	Right	Ankle pain and instability	Increased ROM STJ, instability	Sprain	1.2	X-ray, CT, MRI	TB, sclerotic irregular margins	2.8 × 1.1 × 0.8 cm	Excision	Pain and instability improved	0.5
Thiel [8]	2010	12	M	Right	Intermittent dull ankle pain after heavy exercise	FROM, tenderness on palpation talus	No	2	CT, MRI	TB, cystic, sclerotic areas, BME	Posterior 1/3	Fixation, 1 screw	Asymptomatic, participating in sports	0.75
Mann [11]	2010	14	M	–	Ankle pain	25% restricted ROM TCJ	No	–	CT	TB	20–25% post STJ	Excision	Functional recovery after 4 mo	2
		13	F	–	Ankle pain	25% restricted ROM TCJ	No	–	CT	TB	20–25% post STJ	Excision	Limited recovery after 9 mo, no sports	1.5
		15	F	–	Ankle pain	–	No	–	CT	TB, flattened talar dome	20–25% post STJ	Excision	Functional recovery after 4 mo	5
		16	M	–	Ankle pain during activities	50% restricted ROM TCJ, 50% increased ROM STJ	No	–	CT	TB	20–25% post STJ	Excision	Functional recovery after 6 mo	3
		15	M	–	Ankle pain during activities	25% restricted ROM TCJ	No	–	CT	TB	20–25% post STJ	Excision	Exercise-induced pain	2
Rammelt [13]	2012	31	F	Right	Occasional ankle pain	Restricted ROM	Repetitive sprains	1.2	CT, MRI	TB, displaced fragment	1/3 talar dome, both joints involved	Fixation, 2 screws	Pain-free ADL, FROM	3
		25	M	Right	Intermittent ankle pain during activity	Restricted ROM	No	3	CT, MRI	TB, irregular shape, degenerative arthritis	Posteromedial 1/3 talar body	Excision	Mild pain during exercise, unable to play soccer, no functional restriction	2
		27	F	–	Evaluation ankle sprain	–	Sprain	0.2	MRI	TB	Both joints involved	Conservative	Minimal symptoms	3
		22	F	Left	Increasing ankle pain	–	No	2	CT, MRI	TB, incongruent both joints, degenerative arthritis	–	Excision	No pain ADL, mild pain activity, no progression arthritis	1
Chandoga [6]	2012	30	M	Right	Evaluation after sprain	Swelling	NR	0	X-ray, CT, MRI	TB	–	–	–	–
Rose [12]	2012	11	M	Right	Ankle pain	Restricted ROM STJ, cavovarus deformity	No	0.5	CT, MRI	TB, congruent joints	25% STJ	Fixation, 2 screws	FROM, pain-free, AOFAS 100	4.2
		15		Left	Ankle pain	–	No	–	X-ray, CT	TB, congruent joints	15% STJ	Fixation, 2 screws	FROM, pain-free, non-union (broken screw), AOFAS 100	2.3
		19	M	Left	Ankle pain	Subtalar complaints	No	Several years	X-ray, CT	TB, degenerative changes STJ	25% STJ	Excision + subtalar arthrodesis	RTW, mild pain, AOFAS 75	4.2
		55	M	Right	Severe ankle pain	Painful and restricted ankle and subtalar motion	No	–	X-ray, CT	TB, oblique separation line, degenerative changes both joints	25% STJ	Excision + pantalar fusion, hindfoot nail	Generalized hind and mid-foot pain requiring analgesia, limited ADL. AOFAS 53	5.6
		26	M	Right	Severe hindfoot pain	Stiffness, painful STJ	No	–	X-ray, CT	TB, degenerative changes STJ	20% STJ	Excision + subtalar arthrodesis	Clinical and radiographical fusion	0.3
Serrato [4]	2013	14	M	Left	Pain after sprain	No ROM STJ, pain plantar flexion	Sprain		X-ray, CT	TB, horizontal talar fracture	Both joints involved	Fixation, 1 screw	No pain	1

*F* female, *M* male, (*F) ROM* (full) range of motion, *TCJ* talocrural joint, *STJ* subtalar joint, *TB* talus bipartitus, *mo* months, *y* years, *ADL* activities of daily living, *RTW* return to work, *AOFAS* American Orthopaedic Foot and Ankle Society score. *Age* duration and FU in years

Over the last 15 years, three patients with a symptomatic talus bipartitus were identified in our institution and one had a bilateral talus bipartitus.

## Case descriptions

### Case 1

**Fig. 1:**
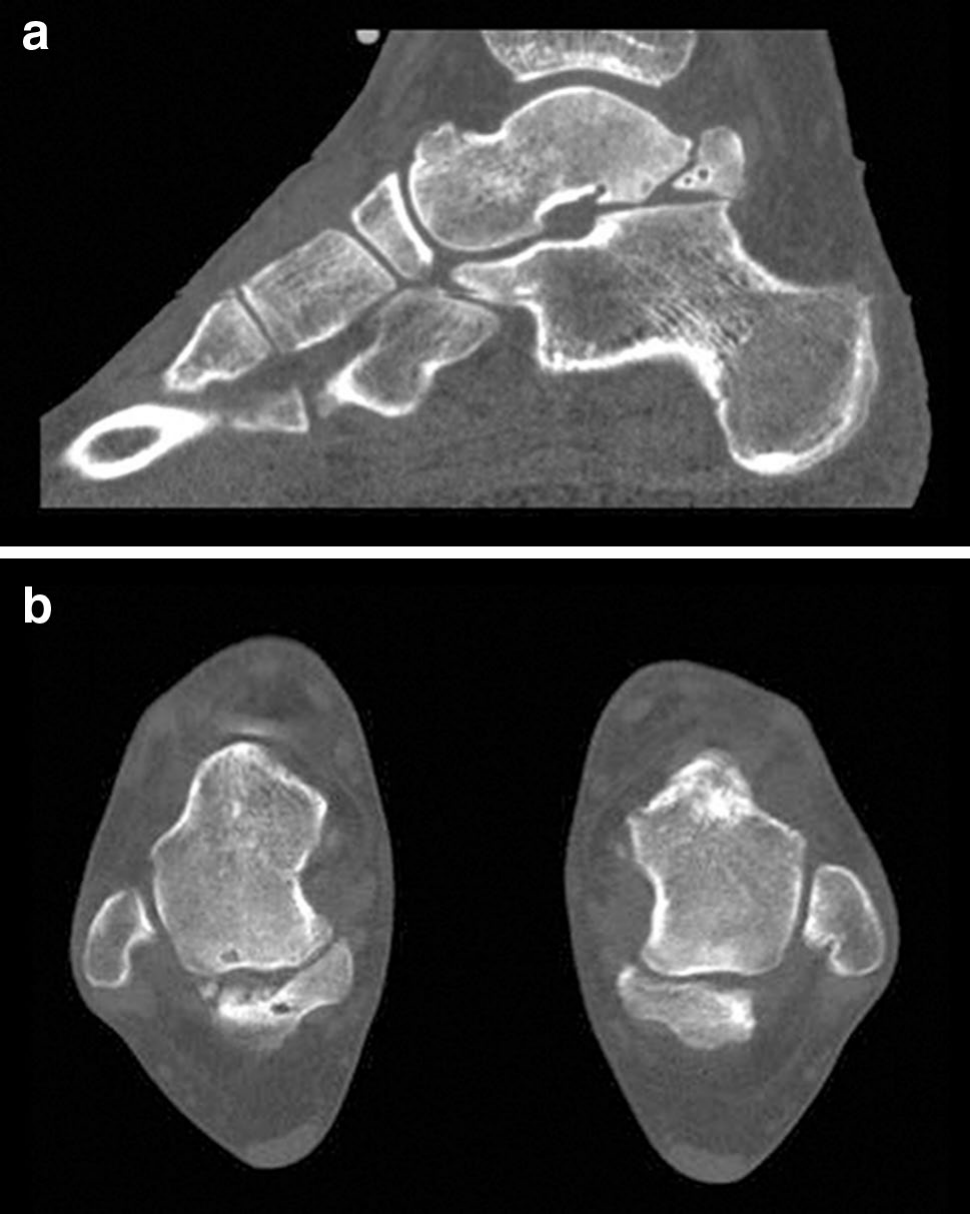
CT scan case 1. **a** Sagittal view of the left ankle showing a talus bipartitus. **b** Axial view showing a bilateral talus bipartitus

A 26-year-old female presented with pain in both ankles for over one year. History revealed recurrent bilateral ankle sprains. Pain was mainly triggered by ankle plantar flexion during walking, specifically when wearing high heels. There were no complaints of instability or swelling after activity. She did not perform any sports activities. Physical examination revealed recognizable tenderness on palpation, especially on the medial and posterolateral aspect of both ankles. Some crepitus was felt over the flexor hallucis longus (FHL) tendon at the level of the ankle joint. The hyper-plantar flexion test was positive bilaterally. Weight-bearing radiographs and the computed tomography (CT) showed a talus bipartitus in both ankles, with early degenerative changes in the subtalar joint, especially at the posterior facet (Fig. 1).

Conservative treatment by means of physiotherapy was unsuccessful. Since the left ankle was most symptomatic, in close correspondence with the patient, it was decided to treat the left ankle surgically by means of an arthroscopic excision of the fragment through the two-portal hindfoot approach [20, 21].

**Fig. 2:**
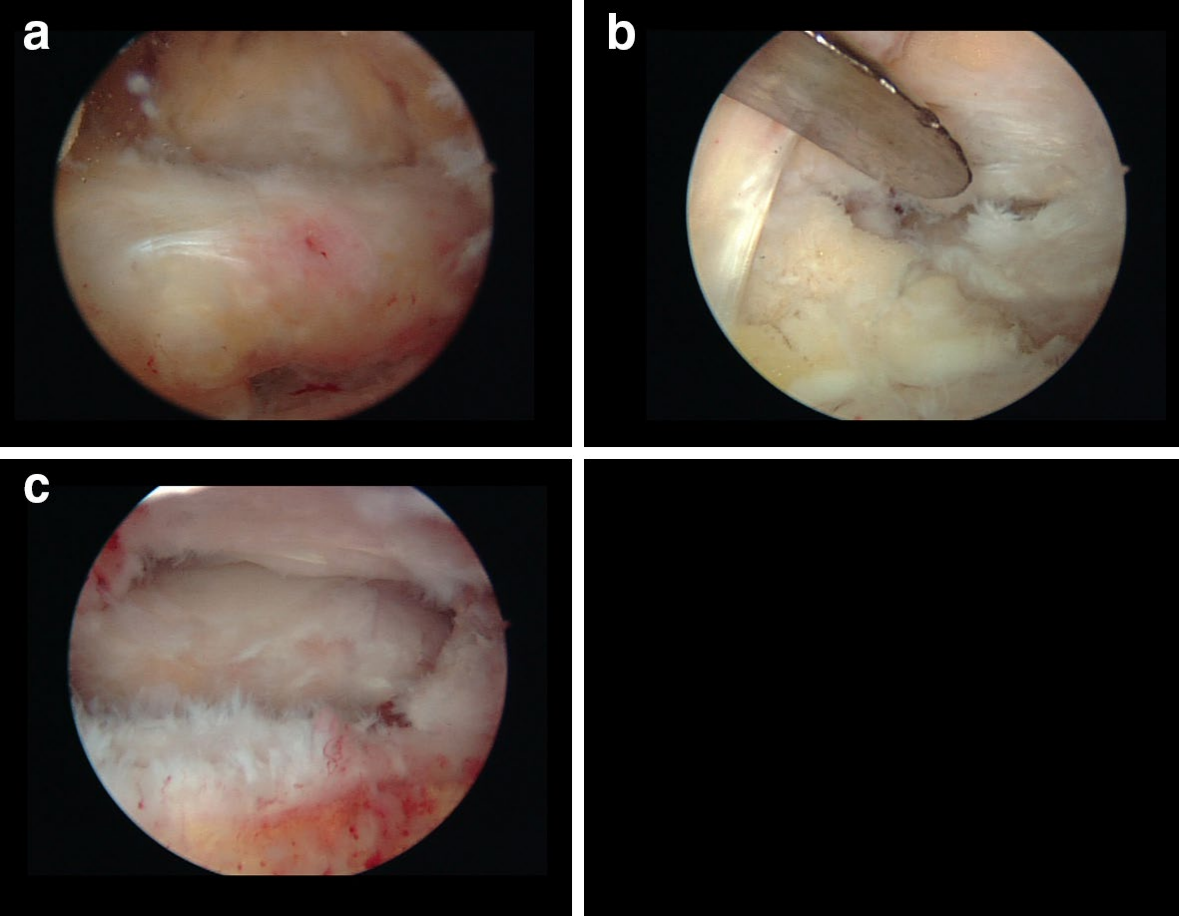
Arthroscopic view. **a** Posterior fragment is identified. **b** Release of the soft tissues surrounding the bony fragment with the periosteal elevator **c**. End result after removal of the posterior fragment

The procedure was carried out in our outpatient clinic under general anaesthesia with the patient in the prone position. Standard posterolateral and medial portals were used. With the arthroscope in the posterolateral portal, the FHL tendon was identified. The posterior bony fragment was released from its surrounding tissues, being the posterior talofibular, talocalcaneal and tibiotalar ligaments and the flexor retinaculum (Fig. 2). Subsequently, the fragment was split into a posteromedial and posterolateral part by means of a chisel to ease extraction through the portals.

**Fig. 3:**
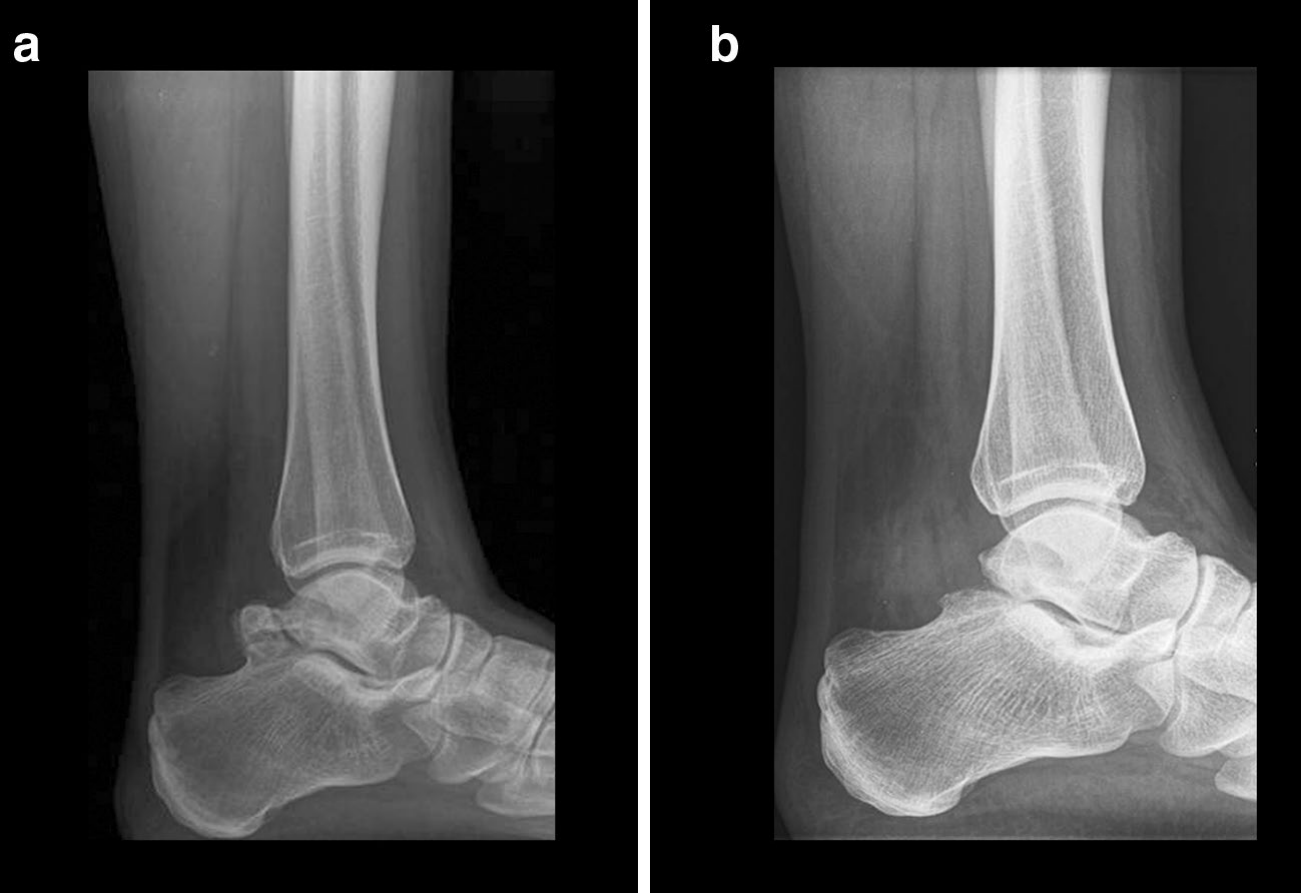
Lateral radiographs case 1. **a** Preoperative radiograph showing talus bipartitus. **b** Radiograph one year following surgery

Postoperatively, the patient was allowed full weight bearing as tolerated. At 6-week follow-up, the patient was free of symptoms and she was able to perform all normal daily activities without discomfort considering the operated ankle; however, the right ankle remained symptomatic. The portals had healed uneventfully and no transient or permanent neurovascular compromises were noticed. At one year following surgery, the patient was satisfied with the results (NRS 10/10) and AOFAS score had improved from 36 preoperatively to 90. The radiographs showed no signs of progressive degeneration at the level of the subtalar joint, as compared to the preoperative situation (Fig. 3). Due to the ongoing symptomatic right ankle, she was keen on having a similar arthroscopic surgical intervention. Two years following the second arthroscopic procedure, she had no pain and was not restricted in any of her activities. At final follow-up, ten years postoperatively, she was satisfied with the result for both her ankles (NRS 10/10), there were no pain issues (NRS 0/10) and she rated her ankle function as being ‘normal’.

### Case 2

**Fig. 4:**
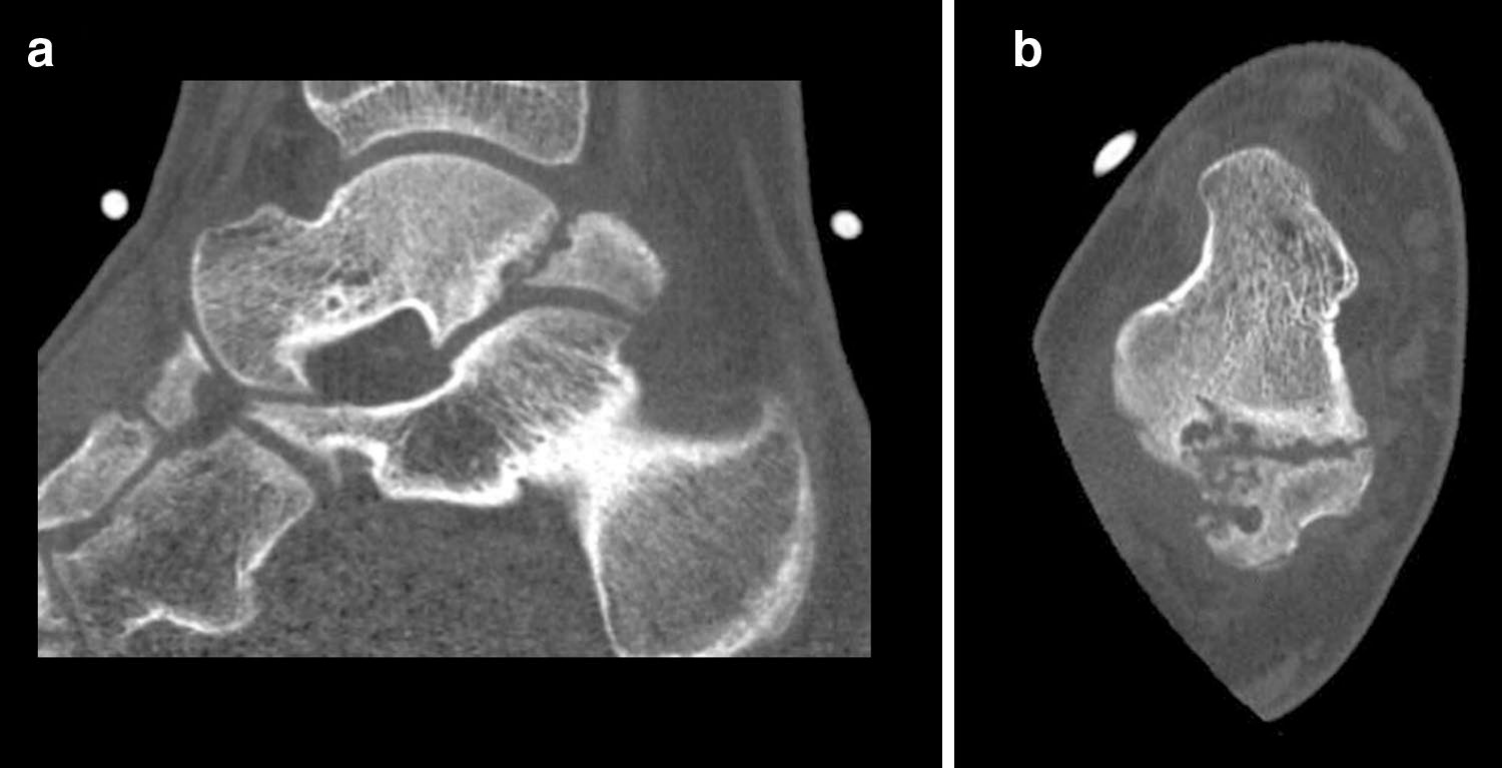
CT scan case 2. **a** Sagittal view indicating talus bipartitus with degenerative changes. **b** Axial view of the right ankle

**Fig. 5:**
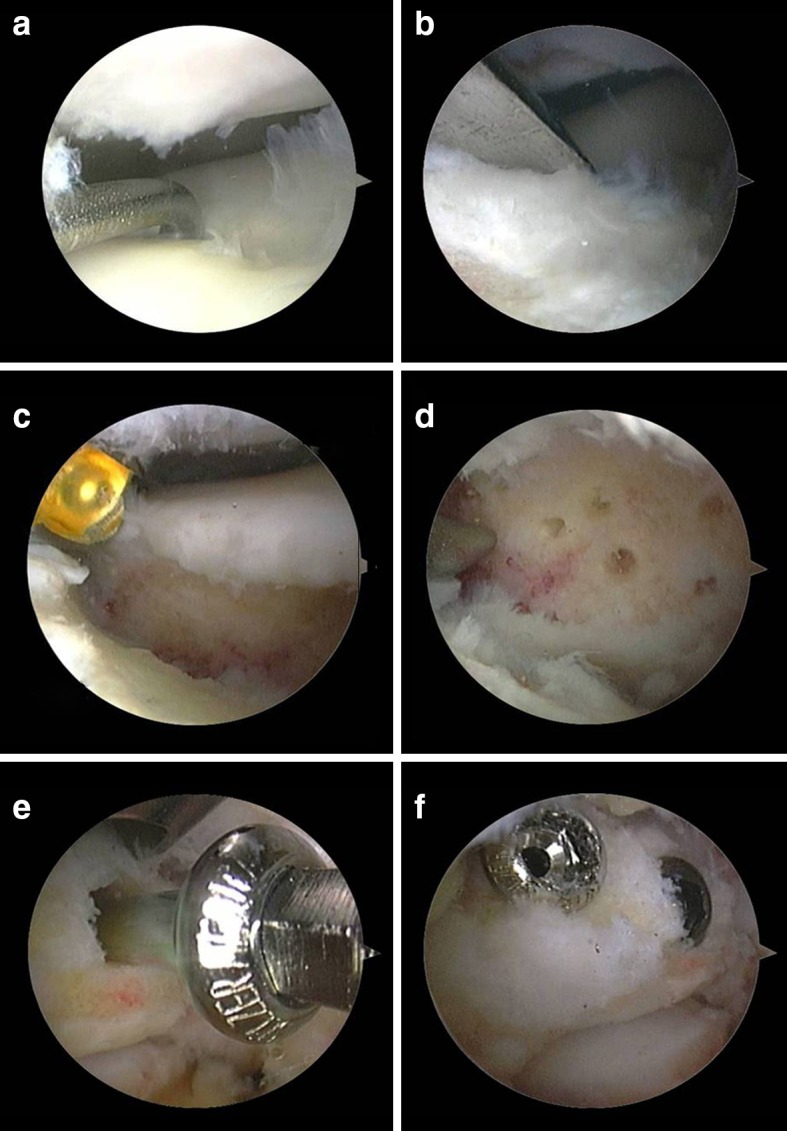
Arthroscopic view case 2. **a** Distinction made between fragments using arthroscopic hook. **b** Splitting of the fragments with periosteal elevator. **c** Debridement of the surface between the fragments. **d** Arthroscopic image following microfracturing of the surfaces. **e** Fragment stabilization performed using two cannulated partially threaded cancellous screws. **f** Arthroscopic view at the end of the surgical procedure

A 18-year-old male presented with posterior ankle pain for 18 months without a preceding trauma. Pain aggravated during physical activity and prevented his participation in rugby. On examination, the right ankle was swollen and was specifically tender posterolaterally. Plantar flexion was restricted by 20 degrees, as compared to the contralateral unaffected ankle. The hyper-plantar flexion test was positive. Standard weight-bearing radiographs and the CT scan revealed a talus bipartitus (Fig. 4). Conservative treatment consisted of physiotherapy, a single corticosteroid injection and immobilization in a cast for 6 weeks. Despite these conservative measures, symptoms persisted with a significant impact on patient’ quality of life and therefore surgery was initiated. Since there was a considerable sized bony fragment, affecting a significant portion of the subtalar joint, it was decided to fix the fragment onto the talar body by means of a screw. Minimal interference with the periosteal tissue during the fixation was achieved by an arthroscopically assisted surgical technique. A standard two-portal hindfoot endoscopy was performed. The fragment was detached by means of a small fragment curved periosteal elevator. The pseudoarthrotic tissue was debrided by means of a curette and shaver. In order to stimulate bone healing, the fragment and talar body were microfractured with a dedicated probe. After adequate repositioning the fragment, fixation was obtained by two small fragment cannulated partially threaded cancellous screws. Postoperatively, the ankle was immobilized in a lower leg cast for 12 weeks, 6-week non-weight bearing and 6-week weight bearing and prophylactic dosages of low molecular weight heparin were given during the entire immobilization period (Fig. 5).

At three-month follow-up, the weight-bearing radiographs showed a good position of the fixed fragment with early signs of union. The patient was allowed full weight bearing as tolerated and physiotherapy was initiated. At 6-month follow-up, the patient had no pain and a full range of motion on physical examination. Patient was allowed to resume his rugby activities as tolerated. At one year after surgery, the patient was persistently free of symptoms and had fully resumed rugby activities. The AOFAS score had improved from 24 preoperative to 91. Patient satisfaction was maximal (NRS 10/10). At final follow-up, 8 years after the surgery, he was still satisfied with the result (NRS 7/10), had occasionally pain after activity (NRS 4/10) and rated his ankle function as ‘nearly normal’.

### Case 3

**Fig. 6:**
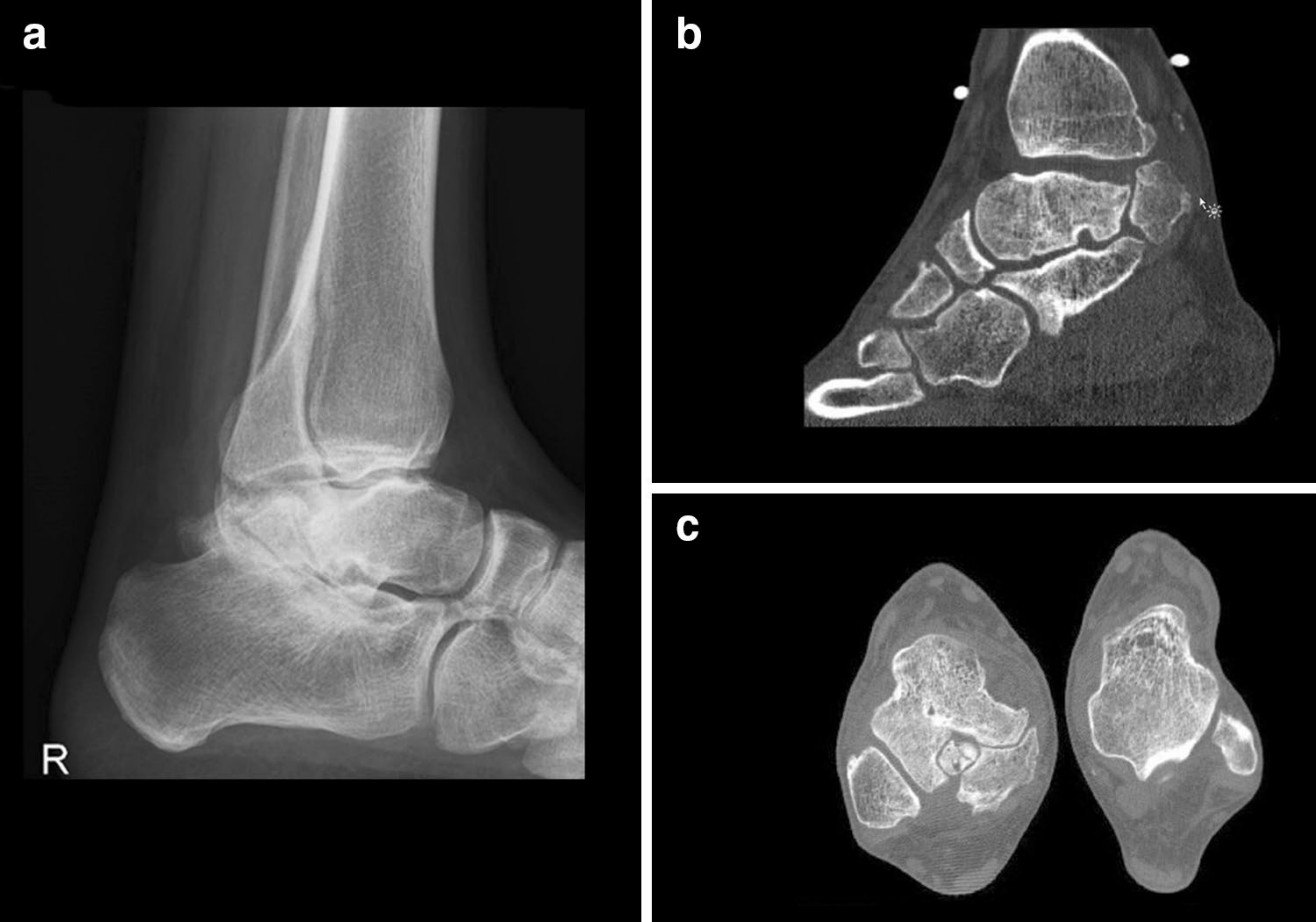
Radiographic images case 3. **a** Weight-bearing lateral radiograph showing a deformed talus and osteoarthritic changes in the subtalar joint. **b** Sagittal CT image showing a flattened talar dome and talus bipartitus. **c** Axial CT image showing a talus bipartitus at the right side. The left talus reveals a normal morphology

A 30-year-old male presented with intermittent but progressive pain of the right ankle during activity without ankle swelling or laxity. There was a history of congenital bilateral clubfeet, for which he was treated conservatively in lower leg casts. Physical examination revealed a stiff hindfoot in varus at both sides and a flatfoot deformity. The weight-bearing radiographs showed a flattened talus. On CT scan, besides the extensive degeneration of both the ankle and subtalar joint, a strongly deformed talus bipartitus was detected (Fig. 6). Due to the limited impact of the complaints on his daily life, it was decided to start with steroid infiltrations in the subtalar joint. These injections were effective, and the ankle remained asymptomatic for several months. Injections were repeated occasionally if the pain aggravated. At final follow-up, 15 years following the onset of symptoms, he was still treated conservatively.

## Discussion

Most important finding of this study was the wide variety in presentation of patients with symptomatic talus bipartitus. The presented manuscript provides an overview of the available literature on talus bipartitus. Patients with a symptomatic talus bipartitus predominantly develop non-specific ankle pain on activity. A limited range of motion was noticed in half of the patients. This condition can remain asymptomatic for at least a couple of years, and two cases were detected coincidently after the follow-up of an ankle sprain. There was a wide variety in shape and size of the posterior fragment. In 38% of the patients, signs of degenerative changes were present, and the extent of these changes depended on the congruency of the fragments in both the ankle and subtalar joints. In the reviewed literature, patients had a median age of 14 years, with a single patient aged 55 years as an outlier. The youngest patient was 11 years old. This is in accordance with the age at which the development of a second talar ossification centre was completed [10].

In the literature, talus bipartitus is also known as ‘talus bipartita’, ‘talus partitus’ or ‘frontal split of the talus’ [1, 15]. It is a rare congenital anomaly, in contrast to the more common and well-known os trigonum. The symptomatic os trigonum often can successfully be treated by Arthroscopic resection [14]. The os trigonum is located at the posterolateral tubercle of the posterior process, whereas a true talus bipartitus involves both the posterolateral and posteromedial tubercles. In addition, in contrast to an os trigonum, the talus bipartitus can also extent into the ankle joint. This extension of the fragment makes it a different entity, in terms of clinical presentation, treatment and prognosis.

The prevalence of talus bipartitus is unknown. Pfitzner investigated 841 feet and reported on the prevalence of anomalies. He did not mention a single case of talus bipartitus [11]. Also Tsaruta et al., in their radiological study on 3460 ankles, did not find a case of talus bipartitus [19]. Together with the three cases presented in this study, we identified 26 patients with a talus bipartitus. Of them, only two had a bilateral talus bipartitus. In addition, Rammelt et al. reported also on imaging of a case in a book chapter [7, 12]. This concerned a 13-year-old boy with ankle pain for more than a year. In this case, excision of the dorsal fragment was performed. Because of the persistence of complaints, a subtalar fusion was performed as a secondary procedure. However, this case was never published separately.

The majority of the cases have been published over the last decade. This was possibly due to advancements in additional diagnostics. CT and MRI are more sensitive to detect a talus bipartitus in comparison with conventional radiographs. Especially in case of the oblique running cleft, it might be missed on plain radiographs. Probably talus bipartitus is underreported in the literature, as it might be mistaken for a non-union of a talar fracture. A thorough history taking should, however, distinguish already between a talus bipartitus and a talar fracture.

Excision and fixation can both be considered while treating talus bipartitus surgically. The surgical preference depends on the size of the fragment and its involvement in surface area of both the talocrural and or subtalar joint. Two authors previously proposed a treatment algorithm. According to Rammelt et al. [12], observation was indicated for asymptomatic patients, for symptomatic patients fixation was preferred in case of congruent fragments, and resection for incongruent fragments. Joint fusion was indicated when signs of progressive osteoarthritis were present. Rose et al. proposed excision of the fragment when less than 20% of the subtalar articular surface was involved and fixation if the fragment exceeded 20% [13]. However, they did not mention how this should be measured. In our opinion, fixation is recommended when the fragment involves the joint surface of both the ankle and subtalar joint. Excision in those circumstances will lead to a decreased joint surface area which might lead to progressive osteoarthritis. In case the talus bipartitus only involves the subtalar joint, the decision to excise or fix is more challenging. Fixation is theoretically preferred over excision; however, the outcome depends on the size of the fragment, surgical skills and preexisting osteoarthritic changes. In our cases, more than 50% of the posterior subtalar joint was involved, as measured in the sagittal plane. The first presented patient was treated with excision, whereas in the second one the fragment was stabilized. Both patients had good short- and long-term outcomes. Therefore, optimal treatment has to be tailored to the patient and depends on severity of complaints, activities of the patient, size of the fragment, joint involvements and the presence of degenerative changes at presentation.

Two cases were presented in which both fixation and excision were performed using an arthroscopic technique. To our knowledge, this is the first surgical description on the arthroscopic-assisted treatment of talus bipartitus. Arthroscopic treatment is preferable over open surgery because of the advantage of shortened hospital stay, rapid rehabilitation and smaller scars. Posterior ankle arthroscopy has been proven to be a safe and effective treatment option for several hindfoot problems [14]. Although we described only two cases, it seems that the two-portal hindfoot approach is also appropriate and safe for the treatment of symptomatic talus bipartitus. In this study, the first long-term outcomes are reported.

The talus ossifies from two ossification centres [8, 10]. Based on a literature study and a histological study about the chondrification of tarsal primordia in human embryos and foetuses, Cihak concluded that the talus is formed out of the *tibiale* and *intermedium*. The *tibiale* forms the corpus tali and the *intermedium* will develop into the posterior process [4]. In case of independent ossification or failure of fusion, this part is responsible for the os trigonum, or possibly also for a talus bipartitus.

A direct traumatic injury is not likely to cause a bipartition of the talus. Eichenbaum hypothesized partition of the talus as a result of traumatic injury during osseous immaturity [5]. However, talar fractures only occur in high-energy trauma, especially in children even higher forces are needed to cause a talar fracture. Therefore, absence of significant trauma rules out a fracture by definition. A possible alternative theory could be that the fusion of the secondary ossification centre with the talar body is prevented by a single or repetitive trauma during adolescence [10]. In addition to the ontogenetic and traumatic causes, also endocrine factors have to be excluded, since an association between hypothyroid epiphyseal dysgenesis and bipartition of hand and foot bones was described [9].

In the third case, the talus was severely deformed as a result of a congenital clubfoot. This patient had bilateral clubfeet, with a talus bipartitus at the right ankle which became symptomatic later on in life. It is unknown whether there is a direct relation between the talus bipartitus and clubfeet.

This study has several limitations. Due to the rarity of talus bipartitus, only case reports and small cases series were included. The level of evidence is therefore low; however, this review provides the most comprehensive overview of the available evidence. Only two patients that underwent arthroscopic-assisted treatment for symptomatic talus bipartitus were included. Therefore, it was not possible to compare the results of the arthroscopic approach with results of open surgery.

## Conclusion

 Talus bipartitus is a rare anatomical anomaly. Symptoms are characterized by pain and restriction of subtalar motion in young patients. Surgical treatment can consist of excision or fixation of the fragment. We presented two cases of arthroscopically treated cases of talus bipartitus with excellent short- and long-term outcomes.

## References

[1] Blauth W, Harten K, Kirgis A (1986). Frontal talus cleft–talus bipartitus. Z Orthop Ihre Grenzgeb.

[2] Cedell CA (1974). Rupture of the posterior talotibial ligament with the avulsion of a bone fragment from the talus. Acta Orthop Scand.

[3] Chandoga I, Vajcziková S (2012). Talus partitus. Kazuistika. Acta Chir Orthop Traumat Czechoslov.

[4] Čihák R (1972) Ontogenesis and homologies of human carpal and tarsal components. In: Ontogenesis of the skeleton and intrinsic muscles of the human hand and foot. Springer, Berlin, pp 12–59

[5] Eichenbaum MD, Austin LS, Raikin SM (2010). Chronic ankle pain secondary to talus partitus: two case reports. Foot Ankle Int.

[6] Griffet J, Habre J, Abou-Daher A, El Hayek T (2004). Talus bipartitus. Rev Chir Orthop Reparatrice Appar Mot.

[7] Hamel J, Braun A (1998) Bildgebende diagnostik am Fuss. Fuss: Erkrankungen und Verletzungen Darmstadt, Germany: Steinkopff, pp 14–22

[8] Hoerr NL, Pyle SI, Francis CC (1962). Radiographic Atlas of Skeletal Development of the Foot and Ankle: a standard of reference.

[9] Mann HA, Myerson MS (2010). Treatment of posterior ankle pain by excision of a bipartite talar fragment. J Bone Joint Surg Br.

[10] McDougall A (1955). The os trigonum. J Bone Joint Surg Br.

[11] Pfitzner W (1896) Beiträge zur Kenntniss der Missbildungen des menschlichen Extremitätenskelets. In: Schwalbe (ed) Morph Arb. 6. Jena, pp 245–528

[12] Rammelt S, Zwipp H, Prescher A (2011) Talus bipartitus: a rare skeletal variation. JBJS Case Connect (6): e21 10.2106/JBJS.J.00610 21411696

[13] Rose B, Southgate C, Louette L (2013). Bipartite talus: a case series and algorithm for treatment. Foot Ankle Surg.

[14] Scholten P, Sierevelt I, Van Dijk C (2008). Hindfoot endoscopy for posterior ankle impingement. J Bone Joint Surg.

[15] Schreiber A, Differding P, Zollinger H (1985). Talus partitus: a case report. J Bone Joint Surg Br.

[16] Serrato J, Bennett J (2012). Adolescent fracture of the talus associated with talus partitus. J Surg Orthop Adv.

[17] Strehle E (1928) Über Abnormitäten im Bereich der Tarsalknochen und ihre klinische Bedeutung: mit besonderer Berücksichtigung eines selbst beobachteten Falles einer Talusmißbildung: Oberreuter, pp 5–12

[18] Thiel E, Feibel J, Chorey N, Gorsline R (2010). Bipartite talus: a case report. Foot Ankle Int.

[19] Tsuruta T, Shiokawa Y, Kato A, Matsumoto T, Yamazoe Y, Oike T (1981). Radiological study of the accessory skeletal elements in the foot and ankle (author’s transl). Nihon Seikeigeka Gakkai Zasshi.

[20] van Dijk CN, de Leeuw PA, Scholten PE (2009). Hindfoot endoscopy for posterior ankle impingement: surgical technique. J Bone Joint Surg Am.

[21] van Dijk CN, Scholten PE, Krips R (2000). A 2-portal endoscopic approach for diagnosis and treatment of posterior ankle pathology. Arthroscopy.

[22] Weinstein S, Bonfiglio M (1975). Unusual accessory (bipartite) talus simulating fracture: a case report. J Bone Joint Surg.

